# Repurposing phone booths into COVID-19 sampling stations: medical operator experiences

**DOI:** 10.1186/s12939-024-02113-7

**Published:** 2024-02-06

**Authors:** Martin Schoch, Sunaree Lawanyawatna

**Affiliations:** King Mongkut’s University of Technology Thonburi (KMUTT), Bangkok, Thailand

**Keywords:** Asia epidemiology, COVID-19 pandemic, Prevention and control, Adaptive reuse

## Abstract

This study investigated the repurposing of phone booths into COVID-19 sampling stations and assessed medical operator experiences in twelve healthcare facilities in Thailand. Amidst the pandemic, these facilities became sampling stations as an emergency measure to enhance medical staff protection. The stations, repurposed from decommissioned phone booths, aimed to reduce COVID-19 infection risks among healthcare workers and patients. After 13 to 16 months of use, a review collected user satisfaction information from each facility. Results indicated high user satisfaction in addressing protection concerns and increasing swab testing participation. This study discusses station design, operational characteristics, and social benefits, emphasizing their adaptability in resource-limited settings. Findings underscore the potential for repurposing infrastructure in public health emergencies and suggest avenues for future research in sustainable healthcare solutions.

**Supplementary Information:**

The online version contains supplementary material available at 10.1186/s12939-024-02113-7.

## Introduction

Amidst the COVID-19 pandemic, healthcare professionals from several public health centers in Bangkok and adjacent provinces approached King Mongkut’s University of Technology Thonburi (KMUTT) to develop a sampling station addressing concerns by healthcare workers regarding infection risk protection. As a result, twelve COVID-19 stations, designed by repurposing phone booths, were distributed to twelve health facilities in Bangkok and the surrounding areas. Dealing with underserved communities with limited access to health resources, the healthcare facilities reflect a focus on community resilience in resource-limited settings. This study aimed to collect experiences from the medical operators at these facilities.

These redesigned telephone booth stations provide medical personnel with a safer and less exhausting operational approach, with spatial separations in semi-outdoor conditions and indoor cabins receiving air handling. They offer a low contamination risk, allow adaptation to individual needs, and are easily transportable. These COVID-19 sampling stations were adapted by reusing phone booths from existing national telecom operator stock and provide a resilient, workable solution in an ongoing pandemic where access to materials and labor is scarce. However, sampling station operators occasionally raised questions about the legitimacy and usefulness of repurposing phone booths.

Since the reduction of environmental impact from these repurposed phone booths in COVID-19 sampling stations has already been suggested [28], this research examined functional use, operation, maintenance, and maintenance and related social benefits associated with station use by surveying healthcare operator opinions. The study collected feedback and analyzed sampling station utilization to verify its operational characteristics for COVID-19 sample collection or the handling and placement of the sampling station within the selected site. Additionally, the study explored opinions on the appearance and the adaptive reuse/recycle strategy when repurposing phone booths.

## Literature

During the pandemic, the university repurposed unused telephone booths into COVID-19 testing stations. It equipped twelve healthcare facilities for operational use between May and August 2021, with varying usage levels varying from 13 to 16 months at separate locations. The sampling stations, with a capacity to deal with 50 to 100 people daily, provided easy access to testing for a broader population, particularly those with limited access to healthcare facilities. The design included creating a safe work environment, cleaning ease, flexibility in site use, and compliance with basic infrastructure standards. Modifications incorporated a movable platform, stability rods, door installation, and sealing to ensure air tightness. Acrylic panels replaced front and rear glass sheets for glove access and device installation. Further fittings included a combined air conditioning unit with HEPA filtration, an air blower at the rear, and an intercom for better communication between employees and patients. Strategic placement ensured appropriate social distancing between patients and medical staff, reducing reliance on personal protective equipment and minimizing hazardous waste generation [19].

### Need for COVID-19 sampling stations

In 2020, Thailand, like many other nations, faced the COVID-19 pandemic impact, implementing measures such as travel restrictions, lockdowns, and social distancing initially maintained relatively low case numbers compared to some countries [7]. Periodic spikes in cases prompted targeted measures, with regional variations addressed by modified government strategies [26]. Especially during the initial stages, there was a continuous need to perform sample collection for RT-PCR testing [11]. Extended screening capacity was required when governments and related institutions changed their strategy to broad sampling coverage [33]. Where responses proved slow or inadequate, alternative organizations and individuals helped with impromptu solutions.

Many of the adopted testing units were typically comprised of improvised installations or modified structures in or next to hospitals [33], based on media coverage concerning sampling stations used in South Korea [4, 20]. These efforts also included drive-through testing centers, enabling individuals to be tested in their cars [12], and mobile pop-up testing sites established in high-demand locations [6]. Using such semi-outdoor solutions effectively reduced contamination risk and optimized medical resource allocation, highlighting the importance placed on conducting testing in separate locations to prevent overcrowding in healthcare facilities and protect healthcare workers and citizens [14].

In Thailand, utilizing sampling stations from repurposed phone booths arose from the emergent need to offer and implement solutions quickly [30], with decommissioned units available from national telecommunication providers [34]. While early attempts involved cutting holes in the glass or plexiglass panel to attach arm-length rubber gloves that allowed medical staff in PPE to collect patient samples, design progression led to improving airtightness, installing air conditioning, and integrating HEPA filters and fans to maintain controlled conditions for healthcare workers. As a cost-effective solution, they enabled wider distribution and improved public health outcomes by helping to normalize testing, reducing the associated stigma, and making it a routine part of public health [3].

### Social benefits

Commonly understood, social benefits describe an increase in social welfare resulting from an implemented action [29]. In this research, the investigated social benefits refer to improving the conditions for healthcare workers and citizens affected by implementing COVID-19 sampling stations. As such, these benefits primarily focus on healthcare-related effects. COVID-19 swab station usage provided social benefits by offering safer sample collection environments for healthcare workers and patients, protecting them from infection [15], and reducing the need for personal protective equipment [2].

Physical distancing measures implemented in sampling stations can prevent the spread of disease [16, 18] and can be improved by positioning them outside hospitals [20]. The WHO [36] provides guidelines for designing and planning separate treatment facilities that prioritize strict physical distancing and air control measures, including natural ventilation in semi-outdoor areas. Air treatment, including air quality control and HEPA filtration, is also critical in preventing the virus from spreading [5, 9].

COVID-19 sampling stations can improve working conditions and minimize healthcare workers’ (HCW) stress and anxiety while reducing hazardous PPE waste [24]. In highly infectious situations, it is imperative to create suitable sampling stations with cabin enclosures that build up air pressure [22] to ensure safety. Such conditions allow medical staff to conduct the sampling process in an inexpensive, comfortable space while preventing contaminated air from entering the cabin. The cabin enclosure should also be suitable for medical staff to stand in [32], similar to a clean room concept [10].

A further focus is on the described approach to repurposing phone booths and the perception of their utilization in the medical field. The COVID-19 sampling station design provides a safe sample collection environment while demonstrating resource efficiency by repurposing phone booths that are difficult to recycle economically [17, 27]. In addition, adaptive reuse strategies promote sustainable thinking and reduce environmental impact [13, 31]. The long-lasting and variable use of the sampling stations can improve future pandemic preparedness, and post-pandemic use could extend the life cycle [24].

The design also fosters resilience by providing alternative production and material usage options that evade pandemic-related disruptions [1, 25]. The nostalgic appearance and welcoming color scheme associated with the phone booth reduce anxiety about the screening process and support a less stressful healthcare environment than conventional medical facilities [35].

### Post-implementation review and survey

Conducting a post-implementation review (PIR) is crucial to assess a project’s achievements and quality, identify areas for improvement, and document lessons learned for future projects [21]. PIRs involve assessing project objectives, identifying post-change adjustments, and updating lessons learned and ongoing benefits [8]. Using surveys in design studies can advance scientific understanding in socially relevant areas or spatial use studies [37]. PIRs and surveys may have limitations, such as respondents not being forthcoming or survey questions not capturing all project impact aspects [23]. However, they remain valuable tools for evaluating project success and identifying areas for improvement.

## Methodology

**Fig. 1:**
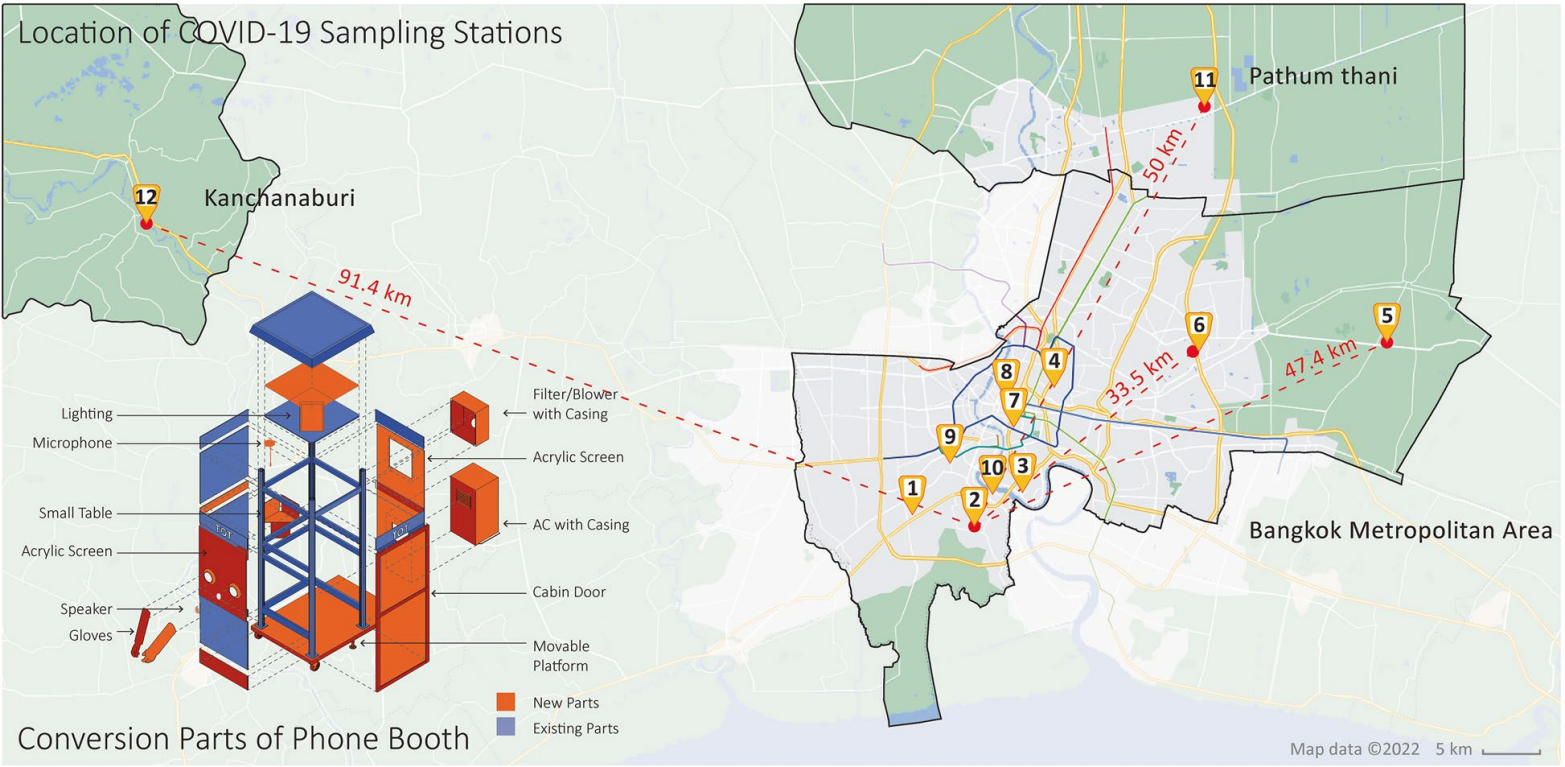
Location Map for distributed COVID-19 sampling stations and a component installment illustration

This study involved conducting a PIR survey among medical personnel using COVID-19 sampling stations made from repurposed phone booths, using a qualitatively oriented questionnaire to reconfirm original design objectives and learn about experiences over the period mentioned the stations were in use. Figure 1 provides a distributed location overview, consisting of ten district health centers directed by the Bangkok Metropolitan Administration, a provincial hospital in Kanchanaburi province, and a health center in neighboring Pathum Thani province.

The figure further shows modifications to the phone booths, including a movable platform, reinforced structure, and acrylic panels for glove openings and mounts. The booths also feature a sealed cabin door, microphone-speaker system, air conditioning, HEPA filter, and blower, providing a safe and compact space that meets medical facility requirements.

### Survey method

The survey focused on collecting experiences regarding the station’s usability and collected feedback from the assigned medical staff at the respective facilities operating as COVID-19 sampling stations. The survey was aimed primarily to include healthcare professionals who were actively involved in collecting and examining patients or who were responsible for its implementation and had supervised. In one case, a facility manager who actively participated and overlooked sampling was permitted to participate. In addition, all participants had to be able to complete the survey in Thai and be willing to answer follow-up questions by phone if needed.

The study used a cross-sectional survey design to collect data from healthcare professionals in twelve healthcare facilities employed for a minimum six-month operational period. The survey assessed various aspects associated with the COVID-19 sampling stations, including user satisfaction, effectiveness in reducing infection risks, and perception of the design approach. The review aimed to find answers in three categories: a) design and site implementation, b) operations, maintenance, and repairs, and c) overall benefit, acceptability, and future use, as outlined in the following:

- Design and Implementation: Concerning the design execution, interest points related to practical use when repeatedly taking samples. For instance, whether the design was sufficient and what problems in using the station concerned design decisions. Another concern focused on actual experiences when installing the station and the number of later relocations to understand flexibility and mobility effectiveness when installing them at various locations.
  Another part focuses on whether user experiences and changes in the evolving pandemic changed the standard operation process. Finally, to some extent, the facility operatives’ resilience mindset was also investigated based on medical operatives’ decisions to compensate for eventual shortcomings in the governmental emergency response and whether to decide on third-party support.
- Operations, Maintenance, and Repairs: This section asks about current utilization in each healthcare facility to determine station usage, sampling procedure implementation, and operation times. Querying usability and performance permitted an examination of their flexibility and suitability. In addition, by looking at the respective district’s land use specifications in conjunction with the case numbers during the crisis peak, information can help identify possible differences in support that different groups may have received. Further, this part examines what problems occurred during their use in terms of operation, maintenance, and repair issues and about finding problematic design elements for future development.
  It also examines whether specific components and devices need unforeseen replacement or repair due to high usage and hygiene requirements. Finally, concerning used parts, the question arises as to whether such an adaptive reuse approach is acceptable in supporting medical procedures, given that medical equipment needs to be reliable.
- Benefit, Acceptability, and Future Use: This section examines respondents’ opinions on general feedback gleaned from sampling station policies, their acceptance of using repurposed phone booths for medical procedures, and their understanding of the benefits derived from reuse. What is of interest here is whether attempts to address environmental concerns were perceived as acceptable or unacceptable, indicating whether such thinking is publicly tolerable, including their position as to independence from existing operations and cooperation channels. Finally, the research aims to identify plans by healthcare institutions to use sampling stations when their original purpose is no longer required.

Data collected through an online application allowed the participants to access and complete the survey via computers or smartphones. The survey was conducted in Thai and translated into English. Anti-bias measures were taken to improve reliability and validity, including pilot testing, unbiased language, random question order, and anonymity. The survey adhered to ethical standards throughout its protocol, starting with the recruitment process, in which survey participants provided written consent to publish their responses. Ethical approval was obtained from King Mongkut’s University of Technology Thonburi (KMUTT-IRB-2022/0912/244).

The survey combined data input, ratings, and closed and open-ended questions for clarification. Accordingly, the requested data input focused on operational facts with the sampling station. Close-ended questions helped identify specific aspects when using COVID-19 sampling stations. At the same time, Likert ratings rated agreement or disagreement levels with questions or statements on a scale from 1 to 5. Open-ended questions allowed participants to provide brief comments as needed at the end of each survey section. All data collected was anonymized during analysis to ensure confidentiality.

### Sample selection and size

All twelve healthcare facilities receiving the test station were invited to submit one survey response from one person actively collecting samples, examining patients, or supervising these activities. In a broader context, however, this only represents a small sample size. Determining a sample size based on all current health centers in Bangkok would require a larger sample size to achieve an appropriate confidence level and margin of error. Therefore, while the survey results are academically credible and informative, they should be regarded as indicative rather than conclusive.

## Results

**Fig. 2:**
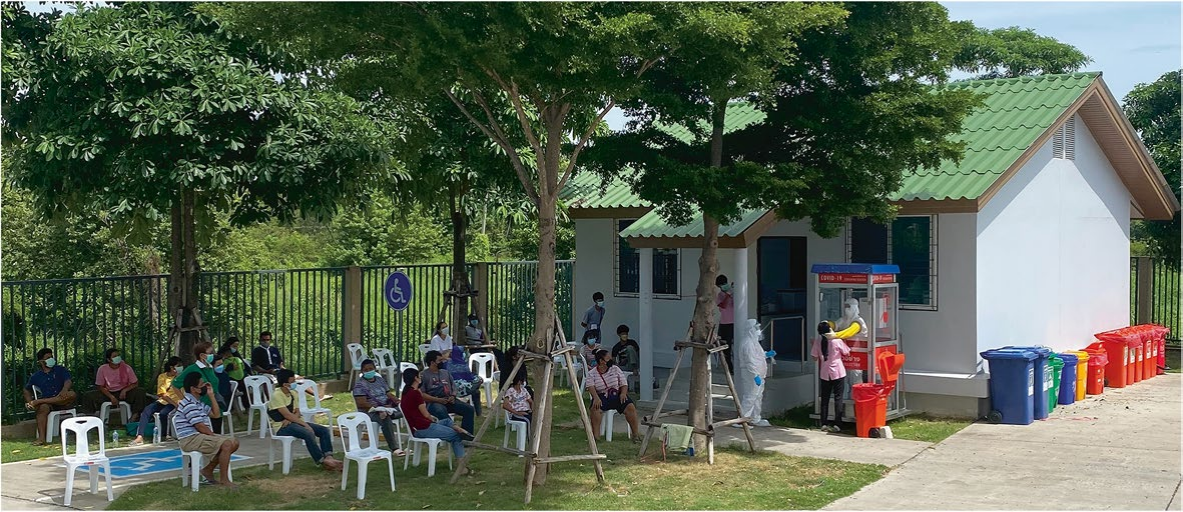
COVID-19 sampling station in use

The study included all twelve locations considered COVID-19 sampling stations. All twelve healthcare facilities agreed to join the survey, and each submitted a valid response about its station usage. The presented results followed the categories described in the methodology. Figure 2 shows an installed COVID-19 sampling station in operation at a medical facility in Bangkok.

### Site implementation and design

The study yielded several positive outcomes. The station design was highly satisfactory for users, with most respondents (91.7%) finding the semi-outdoor environment and safety features (air conditioning, HEPA filters, positive air pressure machines) comfortable. Respondents prioritized medical staff and patient protection as the primary benefit associated with the repurposed phone booths, with 83.3% observing fewer infections in their working team. They agreed that the sampling station significantly improved safety and working conditions and reduced PPE use.

**Fig. 3:**
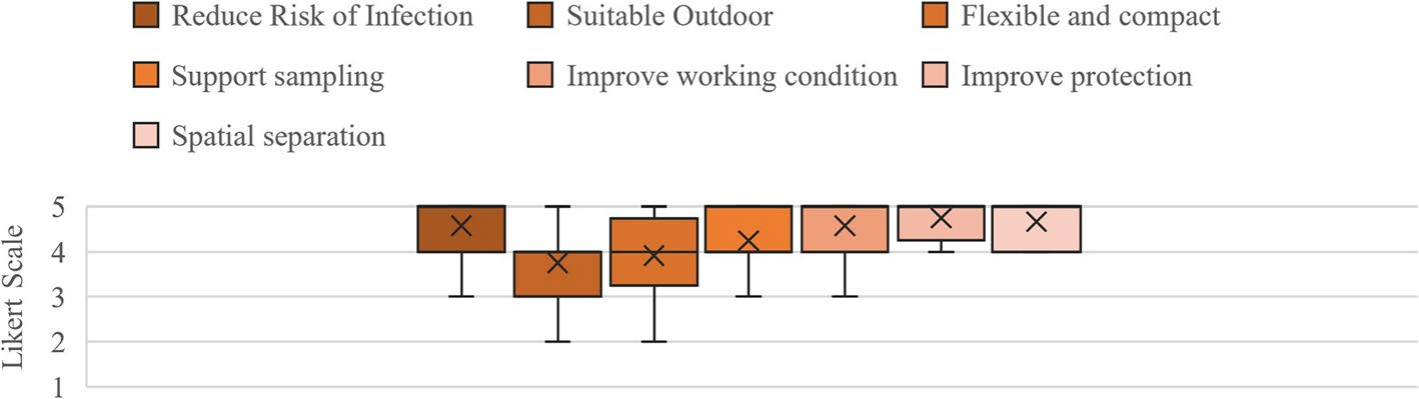
Likert scale survey results (Arithmetic Mean and Standard Deviation) concerning site implementation and design considerations (*N* = 12)

Figure 3 shows that the respondents’ satisfaction level were consistently above neutral on the Likert scale, with slight sample variation in response.

### Operational use, maintenance, and repairs

Table 1 overviews health center locations in city subdistricts and nearby provinces, indicating their daily average use. On average, 5 to 6 healthcare workers operated the stations for about 4 to 5 h daily. The outpost locations provided higher overall service than the district-located sampling stations. The nearby rural province recorded the highest performance, with 375 samples per day. In contrast, city districts recorded an average of between 100 to 200 samplings. This number was more than the initially expected 50 to 100 samples daily. The data shows staffing levels at COVID test points varied moderately, while stations exhibited consistent daily usage. However, there is substantial variability in sampling numbers per day across various locations, highlighting potential disparities in demand or resources. The results indicate that sampling station use in rural land areas was more frequent. Understanding this variance can guide future resource allocation and strategic planning, focusing on locations with higher needs that may require attention to ensure more equitable health service delivery.

**Table 1:** Overview of the monthly average and daily peak usage of COVID-19 sampling stations (*N* = 12)

	***Average assigned healthcare workers per day***	***Average station usage per day***	***Average number of persons per day***
*Average*	*5*	*4h 45min*	*68*
*Median*	*4*	*4h*	*35*
*Maximum*	*10*	*12h*	*375*
*Minimum*	*2*	*3h*	*5*
*Standard Deviation*	*3*	*2h 42min*	*107*

**Fig. 4:**
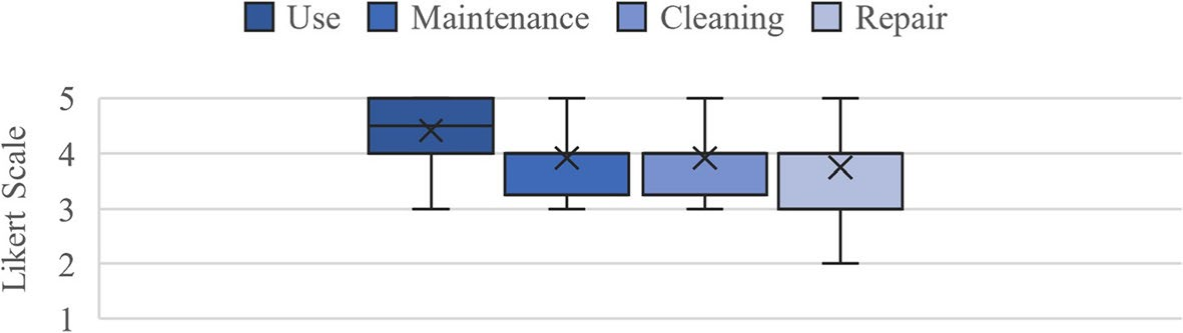
Survey results regarding the sampling stations’ operation (*N* = 12)

Respondents reported that regular disinfection was sufficient for use, maintenance, cleaning, and repair. Some issues were noted, such as damage to the cabin door latch and seal, a blower unit, and a damaged microphone installation. The respondents had mixed opinions concerning the rubber glove quality provided at the station, as they wore out quickly and required regular replacement. Oversized gloves were also challenging but could be solved by wearing matching plastic gloves underneath. Figure 4 shows respondents’ evaluation concerning station operational use, maintenance, cleaning, and repair.

### Overall benefits, acceptance, resilience, and future use

**Fig. 5:**
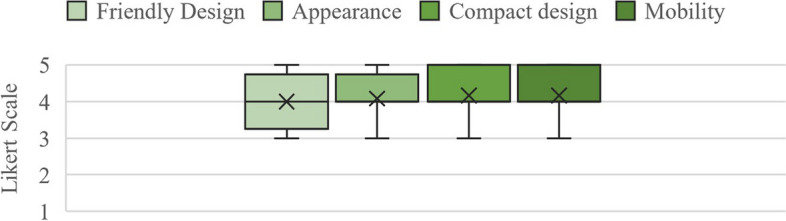
Survey results regarding the sampling stations’ overall acceptance (*N* = 12)

The study also found a high acceptance and support for the adaptive reuse approach of repurposing phone booths for medical purposes. Respondents agree that the stations’ community-oriented and friendly appearance supported COVID-19 testing in underserved areas, and they had no objections or concerns about the sampling stations’ quality, safety, standards, and adaptability. Figure 5 shows the survey results regarding the friendly design, appearance, compact design, and mobility.

The Ministry of Health provided minimal support during the pandemic peaks, leading respondents to seek help from academic institutions. Most respondents (91.9%) believe that relying solely on government support in emergencies is unnecessary and that support from non-governmental organizations that maintain medical standards is acceptable. Although usage declined due to increasing vaccinations and self-testing, most respondents advocate maintaining or repurposing the stations for emergencies, highlighting the sustainable value and adaptability found with the implemented solution. All respondents believe the stations should not be returned or disposed of.

## Discussion and conclusion

This research study examined the effectiveness resulting from repurposing telephone booths into COVID-19 sampling stations employed at twelve healthcare facilities. The focus was to assess their impact on reducing infection risks for healthcare workers and patients. User satisfaction data obtained from healthcare professionals’ experiences were analyzed to understand the design effect. The survey incorporated close-ended and Likert-scale questions, offering a comprehensive view of respondents’ perspectives on station design, operational use, maintenance, and overall benefits. The study’s small sample size was acknowledged, emphasizing the need for further research in a broader range of underserved communities to enhance finding reliability. The investigation stressed the importance of sustainability, community involvement, and unconventional solutions in addressing public health emergencies, demonstrating a commitment to ethical considerations in both design and analysis.

The study demonstrated diverse benefits beyond reducing infection risk. It examined adaptability and feasible alternative uses, notably as healthcare facilities transition from emergency response to an endemic phase. Social benefits were discussed, such as improved working conditions for reducing health worker stress and environmental benefits associated with telephone booth repurposing. The research looked at operational characteristics, ease of use, maintenance, and feedback on repairs, emphasizing the stations’ overall comfort and appropriate dimensions. Capacity utilization data, including daily user counts and site performance fluctuations, were presented.

Respondents accepted repurposed phone booths for medical purposes, with some discontinuing use due to policy changes. In contrast, others favored retention or future repurposing. The study also verified the successful emergency response management in underserved communities and highlighted the importance of community participation, unconventional solutions, and sustainability in addressing public health emergencies. While stressing the resilience of Thailand’s healthcare system, it is suggested that the government should consider promoting emergency responses, investing in community resilience, and promoting sustainable practices in healthcare infrastructure.

Collaboration between healthcare and academic institutions is critical in providing innovative solutions and research support during emergencies. Insights from this study can improve preparedness for future health crises and ensure adaptive and resilient healthcare organizations. The repurposed phone booths are an excellent example of compatible healthcare solutions to meet changing needs. Despite the positive results, the study acknowledges that sample size may have influenced findings, and it is suggested that further research in a broader range of underserved communities is required to enhance reliability. Future research could also evaluate the feasibility of adaptive use in different settings and populations while taking into consideration geographic, economic, and cultural factors. Examining the long-term sustainability of the repurposed structures could also involve assessing their continued relevance and effectiveness for health purposes.

In summary, this research highlights the success of repurposing phone booths into COVID-19 sampling stations and demonstrates positive outcomes such as user satisfaction, operational efficiency, and community acceptance. The study contributes to the literature and emphasizes the importance of innovative, community-oriented solutions to public health challenges, particularly in resource-limited settings. The implemented solution and its sustainable value and adaptability were evident, with recommendations for continued emergency use or further repurposing.

### Supplementary Information

**Additional file 1.**

## Data Availability

The datasets used and analyzed during the current study are available from the corresponding author upon reasonable request.
