# Supplementary material for: Repurposing phone booths into COVID-19 sampling stations: medical operator experiences
Source: Int J Equity Health. 2024 Feb 6;23:23. doi: 10.1186/s12939-024-02113-7 (PMC10848519; doi:10.1186/s12939-024-02113-7)
Supplement: Supplementary file 1 — Additional file 1. [file 12939_2024_2113_MOESM1_ESM.docx]

**Questionnaire and Answers (%) from medical facilities that received the COVID-19 sampling station based on phone booth repurposing.**

Respondents: 12

Usage period: 1.5 years

1. Please describe the location where the COVID-19 sampling station was installed.

8.3% Indoors

0% Outdoors

91.7% Semi Outdoors (Covered)

1. If the location changed while using the station, how often did it change?

58.3% No further change

41.7% One time

0% Two times

0% Three to five times

0% More than five times

1. Please describe the test types for which the sampling work was conducted.

16.7% RT-PCR test

41.7% ATK test

41.7% Both test types

0% Other (Please specify)

1. After the support of the screening station from KMUTT, the medical staff in your institution has a lower number of covid 19 infected from work performance or not.

83.3% Lower

8.3% Same as before

8.3% Affected more

1. Please provide an idea of the usage numbers of the COVID-19 sampling station during

A: Peak Time Use/day

number of the medical staff involved 3-6 Staff

number of screenings 50 / 150-200 / 300-450 person /day

hours of use per day 4-5 Hours

B: Average Time Use /month

Number of the medical staff involved 5-10 Staff

number of screenings 300 / 800 / 2,500 per month

hours of use per day 3-4 Hours

1. Regarding the interior dimension of the station, was the space …

25% fine

0% too small

75% too big

1. Concerning the existing situation for affected healthcare workers at that time, do you think the COVID-19 sampling station improved the following conditions?

100% Improved safety and protection from infection when conducting screening and sample taking.

66.7% Improved working conditions such as thermal comfort or air handling

58.3% Reduced PPE usage

58.3% Ensured spatial separation from possibly infected patients

50% Provided mental support through improvement to prevalent conditions

1. Maintenances and Problematic Part

10% Air filter

90% Gloves

0% Light bulbs

10% Sealings

10% Door lock

1. Resilience mindset and future plan: If the COVID-19 sampling station is no longer needed due to the evolving situation, have you already considered options for the station? Among the suggestions below, which approach would you consider?

58.3% Store for later use

58.3% Alternative use

16.7% Such as Tuberculosis screening

50% Pass to other healthcare facilities

0% Disposal

1. The Acceptance of Circularity Design Concept: Do you think the COVID-19 sampling station based on a phone booth conversion can provide a similar standard of safety and protection as an adequate yet newly built sampling station would have done?

100% Yes

0% No

0% No Opinion

1. Do you feel the university's approach of engaging surrounding communities in the conversion process of the phone booth into COVID-19 sampling stations helped to improve the situation of job scarcity at the time?

100% Yes

0% No

0% No Opinion
